# Capacitance-modulated transistor detects odorant binding protein chiral interactions

**DOI:** 10.1038/ncomms7010

**Published:** 2015-01-16

**Authors:** Mohammad Yusuf Mulla, Elena Tuccori, Maria Magliulo, Gianluca Lattanzi, Gerardo Palazzo, Krishna Persaud, Luisa Torsi

**Affiliations:** 1 Dipartimento di Chimica and CSGI, Università degli Studi di Bari ‘Aldo Moro’, 70125 Bari, Italy; 2 School of Chemical Engineering and Analytical Science, The University of Manchester, Manchester M13 9PL, UK; 3 Dipartimento di Fisica ‘M. Merlin’, INFN and TIRES, Università degli Studi di Bari ‘Aldo Moro’, 70125 Bari, Italy

## Abstract

Peripheral events in olfaction involve odorant binding proteins (OBPs) whose role in the recognition of different volatile chemicals is yet unclear. Here we report on the sensitive and quantitative measurement of the weak interactions associated with neutral enantiomers differentially binding to OBPs immobilized through a self-assembled monolayer to the gate of an organic bio-electronic transistor. The transduction is remarkably sensitive as the transistor output current is governed by the small capacitance of the protein layer undergoing minute changes as the ligand–protein complex is formed. Accurate determination of the free-energy balances and of the capacitance changes associated with the binding process allows derivation of the free-energy components as well as of the occurrence of conformational events associated with OBP ligand binding. Capacitance-modulated transistors open a new pathway for the study of ultra-weak molecular interactions in surface-bound protein–ligand complexes through an approach that combines bio-chemical and electronic thermodynamic parameters.

## Abstract (web summary)

Organic bioelectronic devices can be used to convert biological recognition events to a signal output. Here, the authors show how a water gated field-effect transistor modified with odorant binding proteins can be used to analyse the enantioselectivity of carvone binding and also estimate free-energy changes.

## Graphical abstract

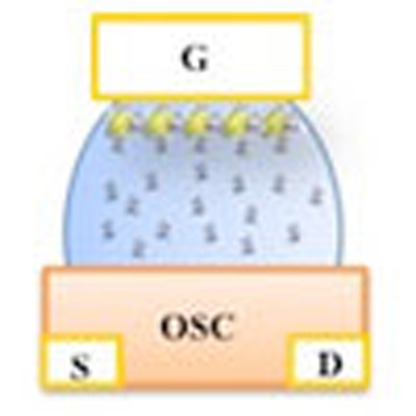

Olfactory transduction is initiated when small volatile compounds (odorant molecules) in air interact with membrane-bound olfactory receptor proteins in olfactory sensory neurons^1^,^2^ after partitioning through the aqueous compartment of nasal mucus of vertebrates^3^ or the sensillum lymph in the antennae of insects. In the mucus or in the lymph small soluble odorant binding proteins (OBPs) are found, which are secreted in high concentrations (millimolar range)^4^,^5^. The binding properties are characterized by a broad selectivity to a range of odorant ligands with dissociation constants (*K*) in the micromolar range^4^. The physiological role of OBPs in the odour perception process is still unclear. They may shuttle odorant molecules from air to the olfactory receptors through the mucus or the lymph, or have a role in the odorant clearance mechanism after the transduction of the olfactory signal^6^,^7^. Recent evidence indicates direct involvement of OBPs in the recognition of different volatile chemicals in insects^8^,^9^,^10^,^11^.

OBPs are extremely stable to temperature, organic solvents and proteolytic digestion. They can be expressed in bacterial systems at low cost, are easily purified^12^ and the binding properties can be tailored through mutagenesis, making them attractive for a number of biotechnological applications. Vertebrate OBPs are characterized by a hydrophobic β-barrel cavity^13^, where the ligand is hosted and the monomeric porcine OBPs (pOBPs)^14^ form a useful model system to study binding affinities to odorant compounds. While pOBPs are weakly negatively charged in pure water^15^, (*S*)-(+)- and (*R*)-(−)-carvone enantiomers are odorant molecules that bear a dipole moment^16^. Understanding of the nature of the ligand–protein interactions at a molecular level is limited, and little is yet known about the interaction of chiral molecules with OBPs.

OBPs have been used as recognition elements in different transducing systems but the reported detection limits do not go below nanomolar level. However, lately bio-detection at very low concentrations has become possible using field-effect back gate^17^,^18^,^19^ and water-gated organic field-effect transistor (WGOFET)^20^ sensors^21^,^22^. Particularly relevant is the case of a back-gate OFET that can provide differential detection of carvone enantiomers at the tens of micromolar concentration level^19^. WGOFETs are operated at very low voltages^23^ and can be fabricated at low cost on flexible substrates^24^. In a bio-FET the gate electrode is generally modified with an electro-active bio-recognition element, and on exposure to the target molecule a threshold voltage (*V*_T_)^25^ shift that scales with the logarithm of the analyte concentration^26^,^27^ has been so far generally measured. The scaling of *V*_T_ with the concentration is a general property of FET transduction^28^ since the reaction electrochemical Gibbs free energy (
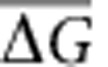
)^29^ changes the electrochemical potential of the gate metal electrons, 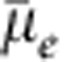. In an ideal trap-free OFET, *V*_T_ defines the gate bias needed to offset the mismatch between the gate metal and the semiconductor electrons electrochemical potential 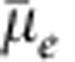. When the reaction takes place at the gate, the 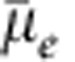 is changed and *V*_T_ shifts. As 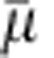 is equal to *μ*+*nFV* (*μ*=chemical potential, *F*=Faraday’s constant, *n*=moles of charges and *V*=electrostatic potential), it is associated with strong long-range coulomb interactions (10–100 kJ mol^−1^). This is why FETs generally provide sensitive detection in reactions that involve charged species, but are less successful for quantitative detection of neutral species^30^. The Gibbs free energy (G) of interactions involving neutral species coinvolves the system energy and entropy, ignoring the effect of net charges or monopoles^31^. *G* can be associated with the interaction energy which accounts for weak shorter-range interactions such as dipole–dipole or dispersive interactions (~2 kJ mol^−1^).

This article reports on the binding of (*S*)-(+)- and (*R*)-(−)-carvone enantiomers to a pOBP-mutant-F88W, detected by means of a WGOFET. With this approach we achieve the sensitive evaluation of energies as low as 1.1±0.5 kJ mol^−1^, associated with neutral enantiomers differentially binding to a pOBP immobilized through a self-assembled monolayer (SAM). The WGOFET output current is governed by the protein nano-layer capacitance where subtle changes can be transduced, with a high signal-to-noise ratio, due to the transistor current gain. Analysis of the free-energy balance and of the degree of capacitance decreases, provides strong support for the hypothesis that on *S*-(+)-carvone ligand binding the pOBP undergoes a conformational change that is not seen with the *R*-(−) enantiomer.

## Results

### Dissociation constants of pOBPs in solution

**Figure 1:**
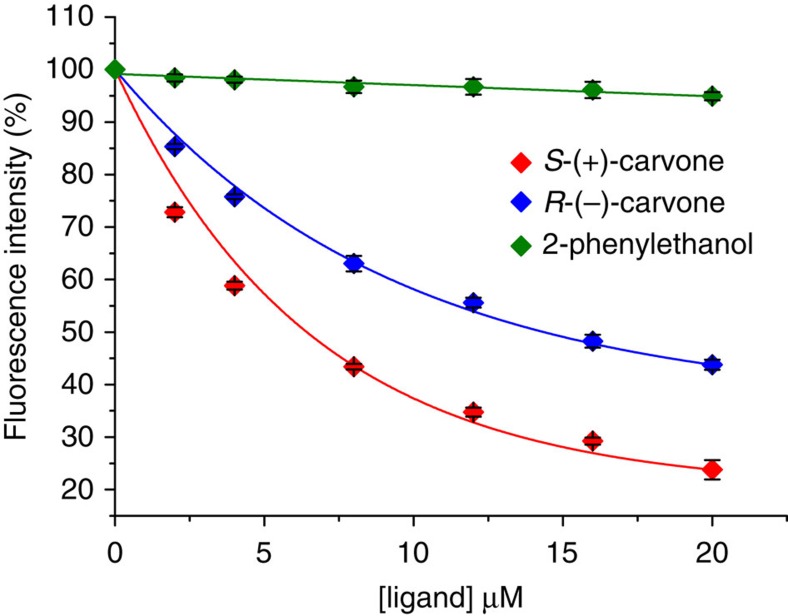
Competitive binding curves for carvone enantiomers and 2-phenylethanol. The affinity of binding of the porcine pOBP was measured using the 1-AMA fluorescence probe. The fluorescent signal is reported as a function of the ligand concentration. A decrease in fluorescence intensity of 1-AMA with increasing concentrations of binding ligands is seen. There is also clear difference in affinities between the carvone and the 2-phenylethanol ligands. Moreover, the decrease in fluorescence intensity of 1-AMA for two enantiomers differs significantly, confirming the differential selectivity of carvone enantiomers by pOBP-F88W.

A competitive fluorescent binding assay performed on pOBPs in solution, returned for pOBP-carvone enantiomer complexes (pOBP-C) dissociation constants *K*_Sol_^+^ of 0.50±0.01 μM and *K*_Sol_^−^ of 1.22±0.05 μM, for the (*S*)-(+)- and (*R*)-(−)-carvone, respectively. The data are reported in Fig. 1 and the details of the dissociation constants extraction are reported in the Method section. These data prove the pOBP-mutant-F88W to be chiral active. The binding is relatively selective, and control experiments indicate that 2-phenylethanol binds very weakly to pOBP (*K*_Sol_~40 μM) (Fig. 1), while wasp-OBP is not capable of differentiation between carvone enantiomers (Supplementary Fig. 1).

### The pOBP-WGOFET functional mechanism

**Figure 2:**
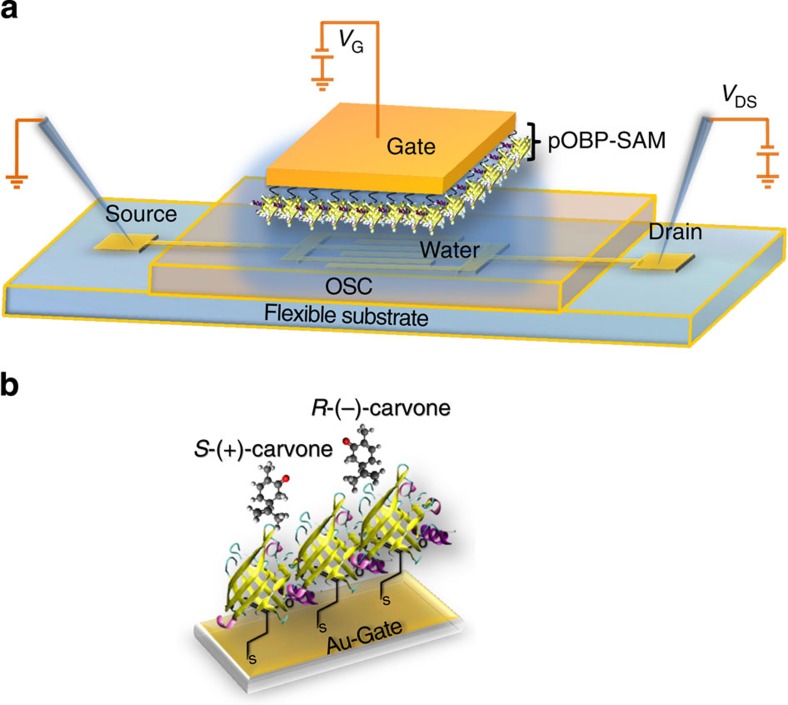
Water-gated bio-organic transistor comprising a pOBP-SAM. In **a**, the WGOFET device schematic structure is shown. The source (S) and drain (D) interdigitated contacts are defined on a flexible foil. The S–D patterned substrate is covered by the p-type, PBTTT-C14 and a 3 μl droplet of water lies on the OSC surface. A bio-functionalized Au-plate hangs in contact with the water droplet, acting as gate (G). The pOBP protein structure is sketched in **b** along with the SAM on the gate surface. pOBP is a monomer of 157 amino acid residues (molecular mass of ~19 kDa) with a height of 38.04 Å and a base of 25.70 × 26.40 Å.

In Fig. 2a, a schematic of the pOBP-WGOFET organic bio-electronic^32^ structure is reported, showing source (S) and drain (D) contacts covered by a spin-coated poly[2,5-bis(3-tetradecylthiophen-2-yl)thieno[3,2-b]thiophene] (PBTTT-C14) p-type organic semiconductor (OSC) deposited on a flexible substrate. A droplet of water is placed on the OSC hydrophobic surface and electrical contact to the gate (G) is made through a gold plate whose water exposed surface is functionalized with a very compact pOBP-SAM, schematically shown in Fig. 2b. An extensive surface characterization of the pOBP-SAM is reported in the Supplementary Information. The data in Supplementary Figs 2 and 3 assess the surface coverage of the gold electrode by evaluating the electrochemical hindrance. In Supplementary Figs 4–6, the SAM surface chemical composition is studied by means of X-ray photoelectron spectroscopy analysis, while in Supplementary Fig. 7 the pOBP-SAM morphology is evaluated by scanning electron microscopy.

**Figure 3:**
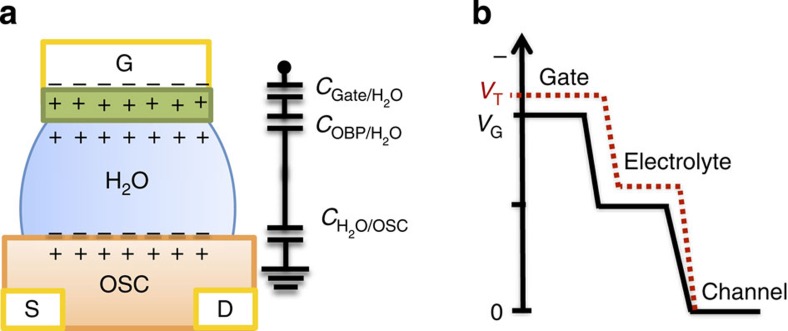
Water-gating mechanism. In **a**, the functioning mechanism of a water-gated field-effect transistor is schematically described along with the capacitors forming the gating systems that are in series. In **b**, the profile of the gate potential drops is reported.

A p-type FET is operated by applying negative *V*_DS_ and *V*_GS_ biases to the drain and the gate contacts while the source contact is grounded (Fig. 2a). Due to self-ionization, pure water becomes an ionic conductor and, on application of *V*_G_, the ions redistribute so that H^+^ ions face the negatively biased gate plate while OH^−^ align at the OSC surface (Fig. 3a). High capacitance electrical double layers at the gate/water, pOBP-SAM/water and water/OSC interfaces eventually form giving rise to the potential drop profile^33^ reported in Fig. 3b. The Au-gate/water and the water/OSC interfaces capacitances per unit area are reported^20^ to be 
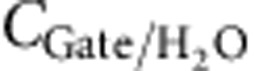
~40 μF cm^−2^ and 
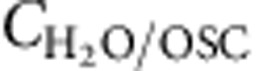
~1–5 μF cm^−2^, respectively. The pOBP-SAM capacitance per unit area (*C*_OBP_) is modelled as a planar capacitor, *C*=*ε*_0_*ε*_r_d^−1^, with *ε*_0_ and *ε*_r_ being the vacuum and the relative permittivity while *d* is the distance between capacitor plates. Taking *ε*_r_=3, typical for a protein system^34^, and *d* as the height of the pOBP-SAM (4.22 nm), a *C*_OBP_=0.63 μF cm^−2^ is estimated. The *C*_OBP_ can be approximated to the whole pOBP-WGOFET gating-system capacitance as this is the smallest in the series (Fig. 3a). This general occurrence is solely connected with the differences in the dielectric constants of a protein-SAM and an OSC compared with water. The actual value of *C*_OBP_ depends, in turn, on several microscopic quantities and parameters.

### The measurements of the electronic responses

**Figure 4:**
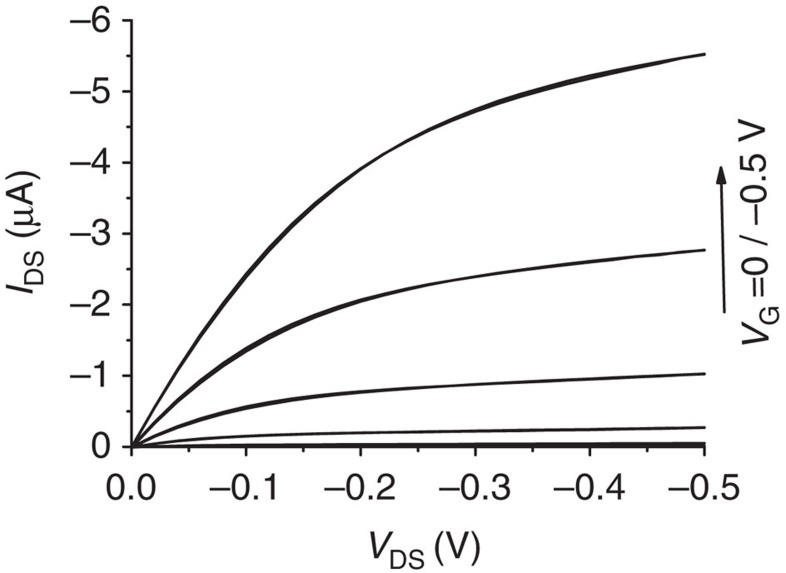
pOBP-SAM WGOFET *I*_DS_ output characteristics. The current–voltage curves are measured in the common-source mode with *V*_DS_ scanned between 0 and −0.5 V. The gate bias, *V*_G_, varies in the same range in steps of −0.1 V.

**Figure 5:**
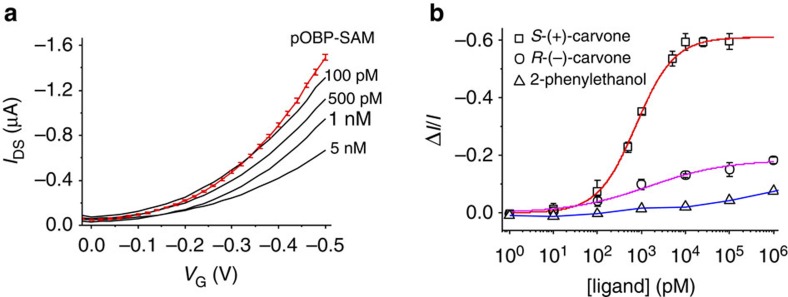
Electronic chiral differential detection of odorant molecules. In **a**, the device *I*_DS_ versus *V*_G_ transfer characteristics are reported both for a pristine pOBP-SAM gate (red curve) and for a gate exposed to concentrations of (*S*)-(+)-carvone in the 100 pM—5 nM range (black curves). These curves were measured after the device stabilization had been performed and an average relative error of 5% is measured for the base-line curve. In **b**, the binding curves gathering the Δ*I*/*I* data points measured with a WGOFET whose pOBP-SAM gate is exposed to the (*R*)-(−)- and (*S*)-(+)-carvone as well as the 2-phenylethanol ligands, in the 1 to 10^6^ pM range, are reported. The data are plotted as the Δ*I*/*I* average values measured on three different devices for each curve.

The applied negative *V*_G_ bias, through capacitive coupling between the gating system and the OSC, induces positive charges in the OSC generating a two-dimensional channel of holes^35^,^36^ (Fig. 3a). As *V*_G_ is applied, the conditions for charge accumulation are set but the actual *I*_DS_ current flow does not start (under an applied *V*_DS_ bias) until *V*_G_ equals *V*_T_ and the energy barrier, originated by the gate electrode and the OSC 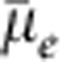 mismatch, is levelled. Beyond *V*_T_, the further injected charges can drift through the channel with a given mobility *μ*_FET_ and output characteristics such as those reported in Fig. 4 are measured. Each applied gate bias (0≤*V*_G_≤−0.5 V) sets the *I*_DS_ maximum intensity with the curves exhibiting the expected linear (at lower *V*_DS_) and saturation regimes while measured at remarkably low biases (*V*_DS_≤−0.5 V). The *I*_DS_ current flowing in the FET channel in the saturation regime is given by^25^:


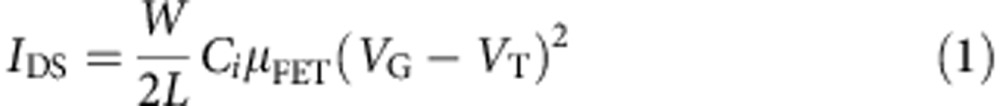


where *C*_*i*_≈*C*_OBP_ is the capacitance per unit area. In Fig. 5a, the pOBP-WGOFET transfer-characteristics (*I*_DS_ versus *V*_G_ at *V*_DS_=−0.5 V) are reported for a device gated with a pristine pOBP-SAM (baseline, red curve) along with the traces measured after exposing the pOBP-SAM to progressively higher concentrations of (*S*)-(+)carvone (signal, black curves). Apparently, a significant degree of current decrease can be measured even at very low ligand concentrations. In the Supplementary Fig. 8, the gate leakage currents (*I*_G_) for the data of Fig. 5a are reported for comparison. These leakage currents are about 3 orders of magnitude lower than *I*_DS_ and do not display a consistent trend with the concentration of the ligand, confirming the need of the FET gain to gather useful responses. From the transfer curve the *C*_OBP_ and *μ*_FET_ product, as well as the *V*_T_ values are extracted^17^. *μ*_FET_ can be as high as (1.1±0.2) × 10^−1^ cm^2^ V^−1^ s^−1^, showing a very good level of performance for the pOBP-SAM WGOFET^37^. The fractional current decrease upon binding was evaluated from the transfer curves as Δ*I*/*I*=[(*I*−*I*_0_)*I*_0_^−1^] with *I* and *I*_0_ being *I*_DS_ values at *V*_G_=−0.5 V for the signal and for the base-line curves, respectively. Δ*I*/*I* was taken as the WGOFET electronic response as this normalizes the device-to-device variation in FET biosensors^38^, resulting in high response reproducibility.

### Enantiomer binding to a pOBP-SAM

**Figure 6:**
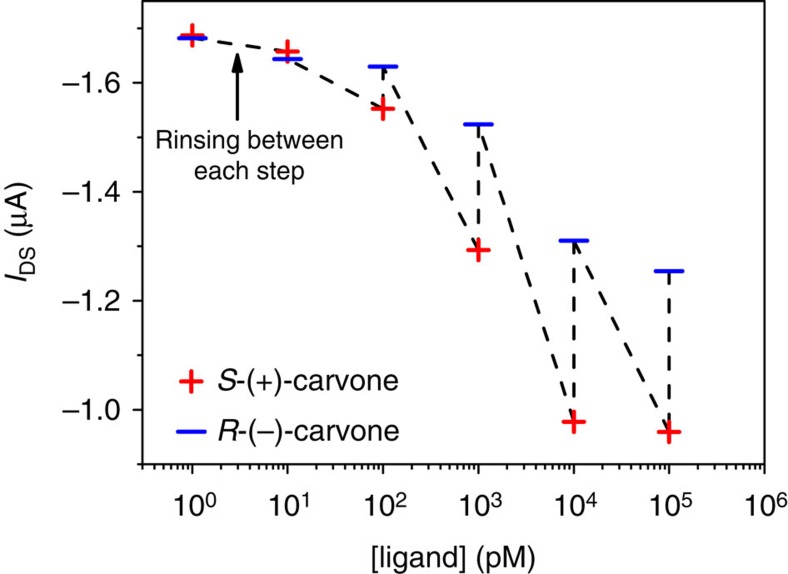
Chiral differential detection of carvone enantiomers performed with the same gate electrode. The *I*_DS_ values measured with a WGOFET bearing the very same pOBP-SAM gate exposed, alternatively, to one of the two carvone enantiomer solutions. Between two subsequent exposures, the gate is rinsed thoroughly. The (+) and (−) symbols mark the *I*_DS_ current values after exposure to the same concentration of either one of the two carvone enantiomers, in the 1 to 10^6^ pM concentration range.

The binding curves of the (*S*)-(+)-carvone, (*R*)-(−)-carvone and 2-phenylethanol ligands to the pOBP-SAM are reported in Fig. 5b as Δ*I*/*I* versus ligand concentration. The curves are distinguishable down to few tens of picomolar concentrations, showing that chiral differential detection can be achieved at extremely low concentrations with a WGOFET. A control experiment, reported in Fig. 6, shows the *I*_DS_ values measured using exactly the same pOBP-SAM gold gate alternating the exposure to the (*S*)-(+)-carvone and (*R*)-(−)-carvone solutions. Between two subsequent exposures the Au-gate plate was rinsed thoroughly with water. The (+) and (−) symbols in Fig. 6 mark the *I*_DS_ values measured at different concentrations of the two enantiomers. The differential detection effect is clearly visible also in this experiment, already in the 10–100 pM range, though to a lower extent compared with the results of Fig. 5b. The *S*-(+)-carvone traces return a maximum fractional decrease of ~60%, this being very similar to the data reported in Fig. 5b. A much higher response at saturation is seen for the *R*-(−)-carvone. This can be explained considering that the memory effect impacts more on the weaker binding species.

The fitting of the (*S*)-(+)-carvone binding curve (red solid line in Fig. 5b) is performed using Langmuir’s isotherm *Y*=*b*_MAX_(*X*/(*K*_FET_+*X*)), where *Y* is the Δ*I*/*I* electronic response proportional, through *b*_max_, to the degree of saturation. *X* is the ligand concentration and *K*_FET_ is the dissociation constant. The fitting of the (*R*)-(−)-carvone (magenta solid curve in Fig. 5b) was calculated using Hill’s binding model, also used to model the data relevant to the 2-phenylethanol (blue curve). The Hill’s equation is *Y*=*b*_MAX_(*X*^*α*^/(*K*_FET_+*X*^*α*^)), with *α* giving the degree of cooperativity (*α*=1 for non-cooperative, *α*>1 and *α*<1 for positive and negative cooperativity, respectively). The fit of the *S*-(+)-carvone data to a Langmuir’s non-cooperative binding isotherm^39^ returns a dissociation constant *K*_FET_^+^=0.81±0.05 nM and a plateau response at a saturation of (Δ*I*/*I*)^+^=0.62±0.01, while the limit-of-detection is 50 pM and the limit-of-quantification is 150 pM. The detection of carvone enantiomers here performed is about 6 orders of magnitude better than the determinations performed with a back-gate device^19^. A comparison between the two systems is not straightforward as they hold different device structures and different recognition elements. However, it is a fact that in the WGOFET a direct interaction between the bio-recognition element layer and an electronic interface, is created. The *R*-(−)-carvone data could only be accounted for by a Hill’s binding isotherm^38^ that, through the *α* coefficient, models the extent of cooperativity of a given binding site. The best-fit parameters, *K*_FET_^−^=20±20 nM, plateau response at saturation of (Δ*I*/*I*)^−^=0.17±0.02 and *α*=0.5±0.1, suggest an anti-cooperative binding for *R*-(−)-carvone. Hill’s equation successfully accounts also for the 2-phenylethanol binding, exhibiting an anti-cooperative behaviour too (*α*=0.3±0.1) but with a much higher *K*_FET_ of 0.7±0.33 μM. The fitting of the *S*-(+)-carvone to the Hill’s isotherm, performed as control, returns a *K*_FET_=0.4±0.2 nM and an *α*=1.1±0.1, consistent with the Langmuir’s fit. The errors are taken as 1 s.d., as for all data presented.

An enantio-selectivity factor (ESF), taken as the ratio between the slopes in the binding curves linear branch^19^ as high as 6.3 is measured here. The binding of the two carvone enantiomers to the pOBP-SAM being characterized by markedly different levels of cooperativity and an exceptionally high ESF, provides evidence that different interactions indeed occur. Chiral detection is a research topic of great relevance^40^,^41^ and, to our knowledge, there has been no previous report showing both differential detection at low picomolar concentrations together with an ESF >6 (refs 42, 43).

## Discussion

**Figure 7:**
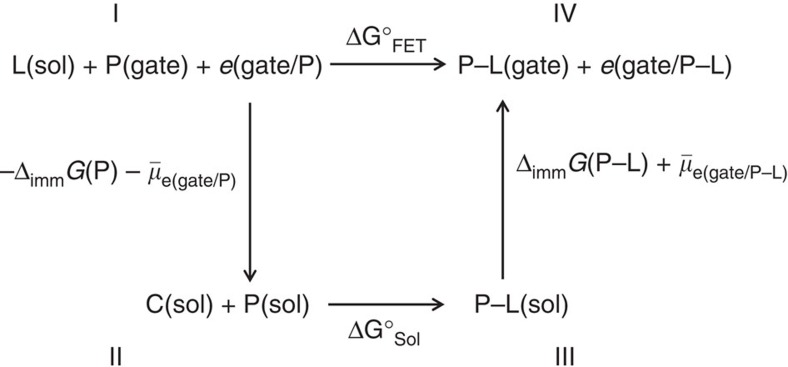
The thermodynamic cycle. It describes the surface and electrostatic effects on the binding equilibrium of proteins immobilized on a gold surface.

All the dissociation constants evaluated with the WGOFET (*K*_FET_) are at least 3 orders of magnitude lower than those measured in solution (*K*_Sol_), although the scale of affinity is preserved. A rationale for the differences in *K* values involves the surface work (*W*) associated with a ligand binding to a layer of orderly immobilized receptors^44^. The change in the metal-gate electron free energy, Δ*E*_F_, also needs to be accounted for. This is opposite to the molar free energy associated to the *V*_T_ shift, so that Δ*E*_F_=−*nF*Δ*V*_T_. Since Δ*V*_T_ is the difference between the *V*_T_ at zero ligand concentration (no binding site occupied) and at saturation (all the binding sites occupied) and *F* being a molar quantity, Δ*E*_F_ is the electrostatic contribution to the molar free energy of the pOBP-C complex formation. The binding equilibrium between a ligand and the pOBP can be written as 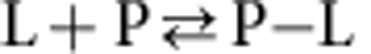, where *L* is the ligand (carvone enantiomers in the present case) that binds to the pOBP protein (P) to give the P–L complex. As the pOBP-SAM is anchored to a gold-gate surface, the equilibrium involves a protein layer/water interface rather than a protein dissolved in solution. The overall interfacial effect can be described considering the thermodynamic cycle reported in Fig. 7. The cycle goes from the immobilized protein P (state I) to the immobilized complex P–L (state IV) involving two intermediate steps. The first step (I→II) concerns the release of the protein from the surface into the solution. The free-energy change associated encompasses the surface work needed to release the protein, that is opposite to the work required to immobilize the protein, Δ_imm_*G*(P), and the change in the gold-gate electron electrochemical potential when the protein layer is removed 
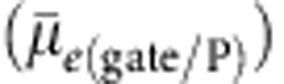
. The second step (II→III) is the binding of L to P taking place in solution with a free-energy change Δ*G*°_Sol_=*RT*ln(*K*_Sol_). The third step (III→IV) is the immobilization of the complex P–L to the gold surface with a free-energy change including the surface work of immobilization Δ_imm_*G*(P-L) and the change in the gold-gate electron electrochemical potential when passing from bare gold to a surface coated with the immobilized P–L layer 
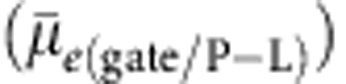
. The fourth step (IV→I) closes the cycle and corresponds to−Δ*G*°_FET_=*RT*ln(*K*_FET_). Following the cycle, the binding standard molar Gibbs free energy probed with the WGOFET, Δ*G*°_FET_, is given by:


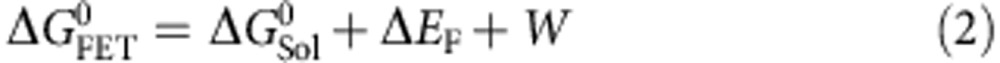


where *W*=Δ_imm_*G*(P–L)—Δ_imm_*G*(P) is the binding surface work term and 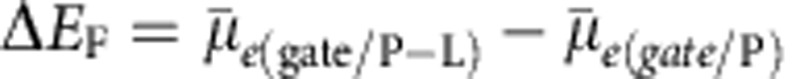 is the change in the gold electron electrochemical potential 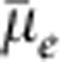 as the gold gate passes from being coated with the P–L to the P layer. Δ*V*_T_=−(Δ*E*_F_/*nF*) is the difference between *V*_T_ at 0.1 μM and zero carvone concentrations. The binding Gibbs energy on a gold gate is split into a molecular interaction and a surface work contribution. The molecular interaction term is associated with the ligand–protein recognition energy in solution. The surface work contribution describes the work spent to accommodate the ligand into a protein bound to a surface. This term (Δ*E*_F_+*W*) includes the conformational rearrangements as well as the change of the gold-gate electron energy.

The binding energy in solution Δ*G*°_Sol_=*RT*ln(*K*_Sol_) is defined as the difference in standard chemical energy between the complex P–L and the reagent partners P and L. The Δ*G*°_FET_ term can be described considering the change in Gibbs free energy between states IV and I. In this case the ligand binding causes the change of physical-chemistry properties of the electrode (including not only the gold plate but also the pOBP-SAM) such as the interfacial tension and the electrons electrochemical potential. The Gibbs energy equation for the system moving from state I to state IV (keeping *T* and *P* constant) is^44^:


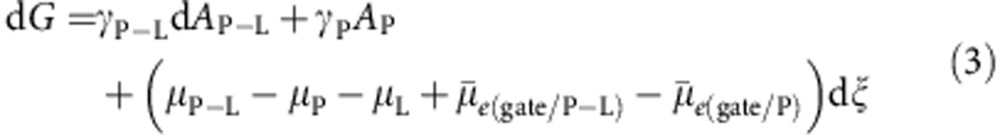


where *μ*_P_, *μ*_L_ and *μ*_P–L_ are the chemical potentials of the protein, the ligand and the complex, respectively; *ξ* is the extent of the reaction variable; *A*_P–L_ and *A*_P_ are the surface areas of the *γ*_P–L_ and *γ*_P_ surface tensions, respectively. The d*A*_P–L_ area increase for the *γ*_P–L_ interfacial phase, corresponds to an area decrease −d*A*_P_ of the *γ*_P_ interfacial phase. Thus, by using the relation d*A*=d *A*_P–L_=−d*A*_P_, equation 3 becomes:


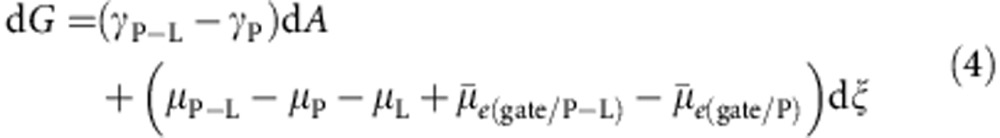


The change of the molar Gibbs energy of the reaction is then obtained by taking the partial derivative with respect to *ξ*:


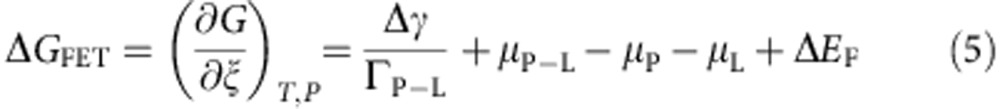


where, Δ*γ*=(*γ*_P–L_−*γ*_P_) and Γ_P–L_=d*ξ*/d*A*_P–L_ describe how the reaction proceeds when the surface is occupied by the complex P–L, that is, it is the surface density of the complex P–L that is constant. By writing the chemical potential as a function of the activity *a*_*i*_ of the *i-th* species and recalling that at equilibrium Δ*G*_FET_=0, equation 5 becomes:


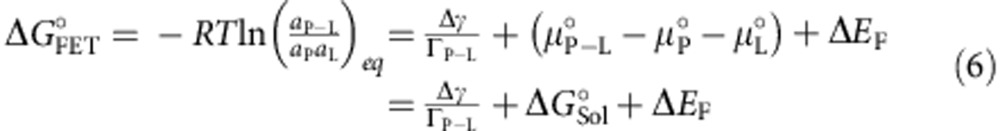


where the activities are at equilibrium and thus the ratio *a*_P_*a*_L_/*a*_P*−*L_ equals the dissociation constant. By identifying the surface binding work *W* with Δ*γ*/Γ_P–L_, equation 2 is fully recovered. The comparison between equations 2 and 6 indicates that *RT*ln(*K*_FET_) is actually the Δ*G*°_FET_ encompassing the molecular recognition in solution, the surface work and the electronic contribution.

From the *K*_FET_^+/−^ values extracted from the data in Fig. 5b, 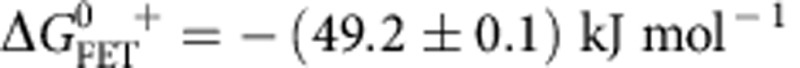 and 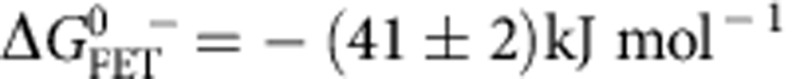 are derived. For the pOBP in solution the figures are: 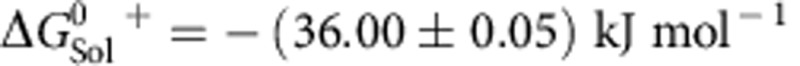 and 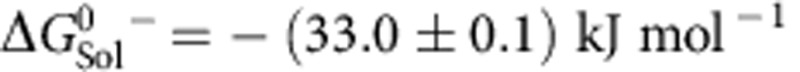. The 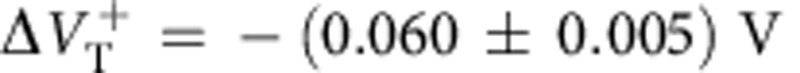 for the S-(+)- and 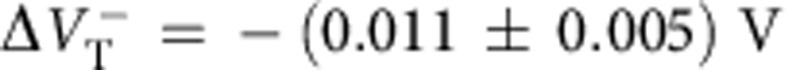 for the R-(−)- enantiomers, result in 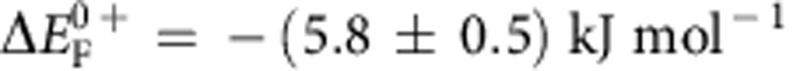 and 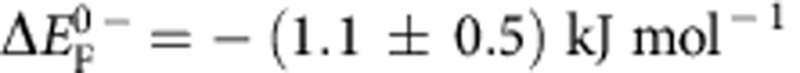, respectively. All the Δ*G*° molar energies estimated so far are shown in Table 1, along with the (Δ*G*^0+^−Δ*G*^0−^) chiral differential values. As 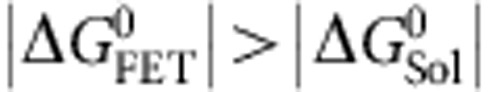 and they are both negative, the binding at the pOBP-SAM is favoured compared with pOBP in solution. This occurrence likely reflects the lower entropy change associated with ligands binding to the orderly immobilized proteins (|Δ*S*_FET_|<|Δ*S*_Sol_|, both being negative contributions). The surface work, *W*, accounts for a fraction of the differences in Δ*G*°_FET_ and Δ*G*°_Sol_ (see equation 2), and since *W* holds indistinguishable values for the two enantiomers (Table 1) the complex immobilization work would be independent from the chiral interaction that involves mainly the pOBP outer cavity. Accordingly, Table 1 shows that the chiral differential binding free energy 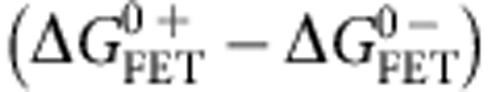 for the immobilized pOBP compares very well to the sum of the 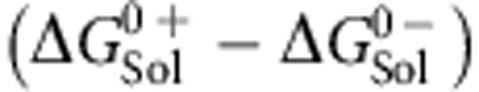 and 
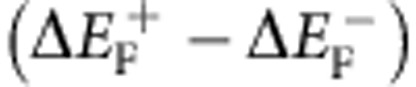
 of independently estimated terms. Such an agreement is remarkable considering that the 
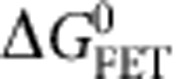
 and the 
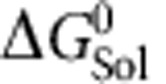
 data are measured under different experimental conditions. Indeed, while with the WGOFET the binding of a ligand to the empty cavity takes place, in the fluorescent assay it is the displacement of a bound reporter ligand that is studied.

Binding curves in Fig. 5b also indicate that the WGOFET response itself is markedly different for the two enantiomers. At saturation, the response to *S*-(+)-carvone is in excess of 60% while it drops to <20% for the R-(−)-carvone. From equation 1 (at constant *μ*_FET_) the response can be written as:


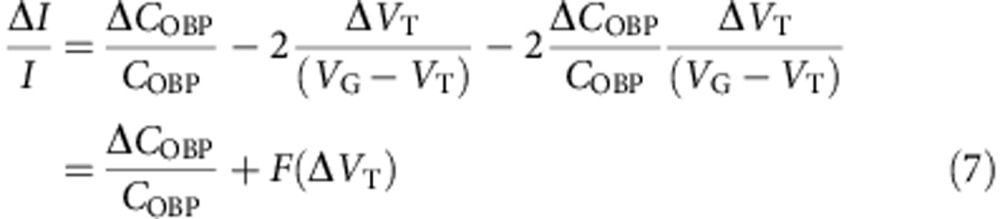


where only the second order terms in Δ*V*_T_/(*V*_G_–*V*_T_) were disregarded. In principle, if |*V*_T_|≪|*V*_G_| and |Δ*V*_T_|≪|*V*_G_| equation 7 becomes:


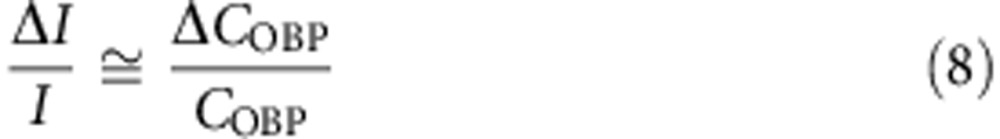


**Figure 8:**
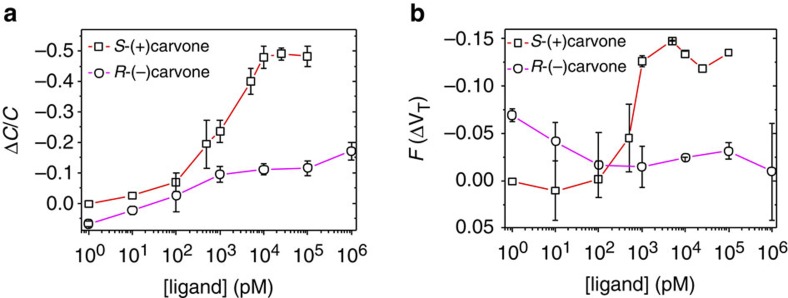
Capacitance and threshold voltage contribution to current fractional changes. In **a**, the fractional changes of the capacitance, and in **b**, the *F*(Δ*V*_T_) term data as extracted from the source–drain current measured on exposure to the (*R*)-(−)- and (*S*)-(+)-carvone ligands in the 1 to 10^6^ pM range.

**Figure 9:**
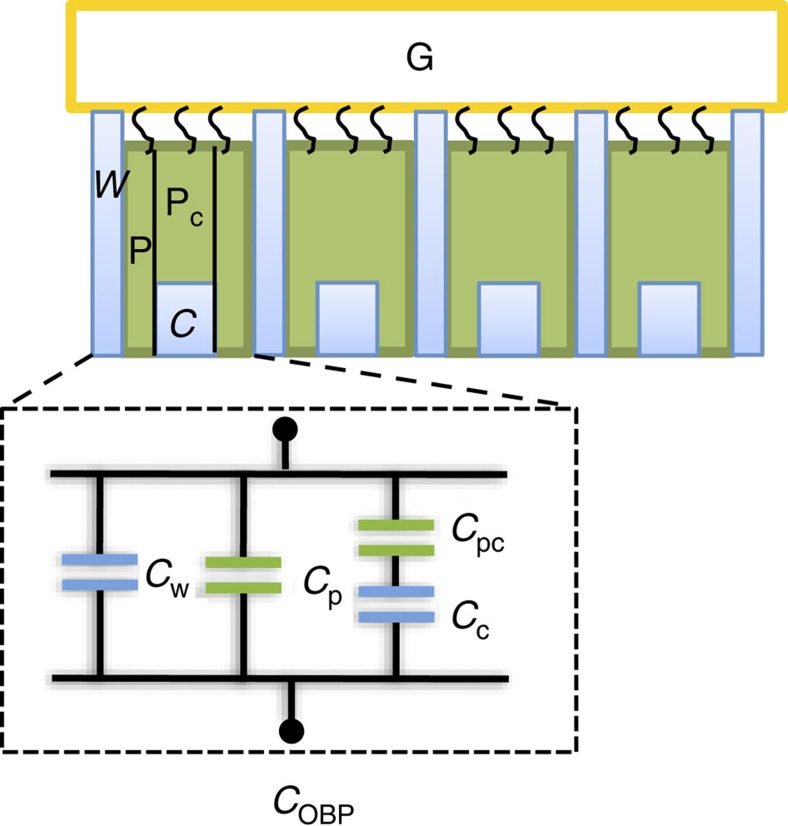
Capacitive-modulated transduction of protein–ligand interactions. The pOBP-SAM layer is detailed as an array of surface immobilized proteins. For the capacitance model each pOBP comprises two plain protein regions (P, P_C_) and a cavity (C). The cavity holds a capacitance *C*_C_, while the protein regions can either be almost as high as the whole pOBP-SAM or be smaller as in the presence of the cavity. These two regions hold capacitances C_P_ and C_PC_, respectively. Contiguous pOBPs are separated by a high dielectric channel, *W*, holding a capacitance *C*_W_. *C*_OBP_ can be represented by the equivalent circuit, formed by the *C*_P_, *C*_C_, *C*_PC_ and *C*_W_ capacitances arranged as reported in the bottom of this panel.

In the present case at saturation, *F*(Δ*V*_T_)≅0.1, while Δ*C*_OBP_/*C*_OBP_≅0.5, as it can be seen in Fig. 8a,b respectively. So, the WGOFET output current is mostly affected by the *C*_OBP_ term. The WGOFET chiral large differential responses are thus associated with subtle changes occurring in the protein upon ligand binding, which lower the pOBP-SAM capacitance to a large extent, as it can be explained through the simple model reported in Fig. 9. The dielectric properties of a protein in the pOBP-SAM layer are described as being characterized by regions with lower (green) or higher (light-blue) dielectric constants. The green parts in Fig. 9 describe the plain protein regions (*ε*_r_=3) while the light-blue ones are the regions characterized by the presence of water (*ε*_r_=80). Indeed the presence of water in pOBP has been already proven^45^. In this model, repeated units are featured that represent the proteins, each one with its cavity filled with water or with the odorant molecule. Each pOBP comprises two different protein regions (P, P_C_) and the elicited cavity (C). The cavity region is modelled with a capacitor, *C*_C_, characterized by a *ε*_r_=80 in the absence of the ligand as in this case water has been shown to be present in the cavity. The cavity *ε*_r_ becomes equal to 3 when it is filled with the carvone. The protein regions, all characterized by the same *ε*_r_=3, can be modelled with a plane plates capacitor having a distance between the plates, *d*, as high as the whole pOBP-SAM (P) or with a capacitor with a smaller *d* due to the presence of the cavity (P_C_). These two regions hold capacitances *C*_P_ and *C*_PC_, respectively. Contiguous pOBPs are separated by a high dielectric percolative path, *W*, addressed as the ‘water channel’ holding a capacitance *C*_W_. The overall *C*_OBP_ can be represented by the equivalent circuit, formed by the *C*_P_, *C*_C_, *C*_PC_ and *C*_W_ elements, reported also in Fig. 9. The capacitors *C*_C_ and *C*_PC_ in series are in parallel with the capacitors associated with the water channel (*C*_W_) and the plain protein (*C*_P_), respectively.

Each different element (P, C and *W*) in the model contributes to the equivalent capacitance per unit surface with weighting factors *α*_p_ for the protein regions, *α*_c_ for the cavity and *α*_w_ for the water channel. These weighting factors correspond also to the ratio between the free surface occupied by a given element and the entire unit, hence: *α*_p_+*α*_c_+*α*_w_=1. Assuming that the cavity occupies approximately one-third of the total protein-SAM (*d*=4.22 nm), the equivalent capacitance per unit surface is:


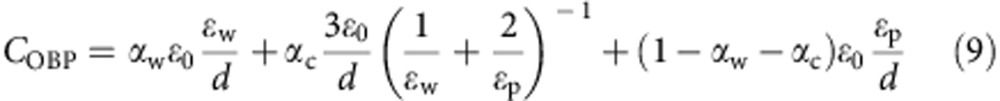


assuming *ε*_w_=80 and *ε*_p_=3, equation 9 becomes:


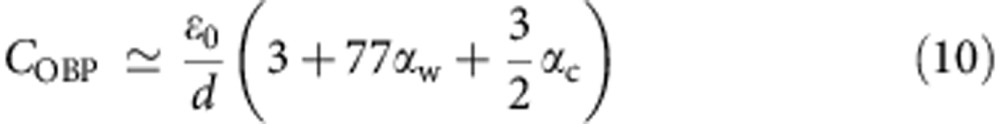


In general terms, the same formula holds also when the ligand (*S*)-(+)-carvone or (*R*)-(−)-carvone is bound. Assuming that the cavity is completely filled by a medium with the same dielectric constant of the protein, we obtain:


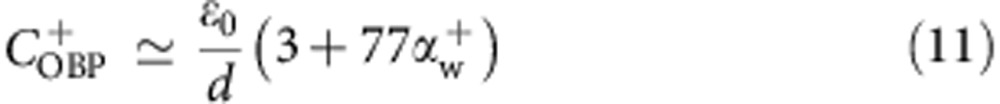



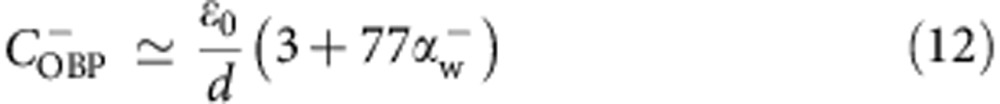


The measured ratios Δ*C*_OBP_/*C*_OBP_ are therefore:


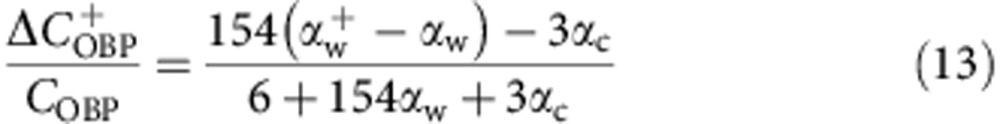



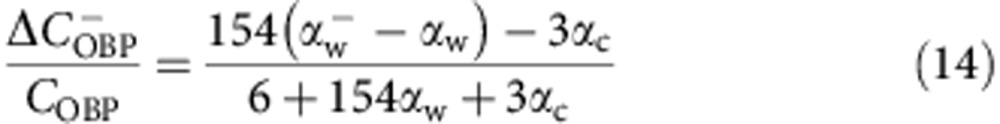


These last two equations were used to fit the measured chiral responses Δ*C*^+^/*C*=−0.485 and Δ*C*^−^/*C*=−0.17.

The model presented contains four unknown parameters, hence some hypotheses are needed to reduce the number of degrees of freedom. From the equations, it is apparent that the crucial parameter is the weighting factor of the water channel that affects the equivalent capacitance with a high numerical coefficient. Since the experimental data show a remarkable difference between the two responses measured for (*S*)-(+)-carvone and (*R*)-(−)-carvone, the fitting had to be carried out assuming that the water channel disappears completely upon binding of the (*S*)-(+)-carvone, while it is largely unaffected when (*R*)-(−)-carvone binds to the hydrophobic OBP cavity. With these hypotheses (corresponding to 
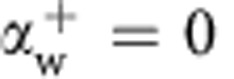
 and 
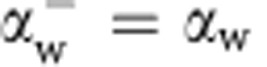
), we can estimate the unknown parameters as:


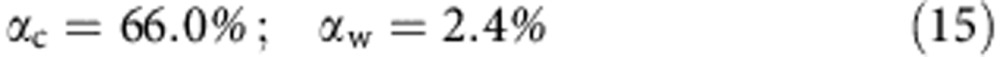


As the high percolative and low *ε*_r_ paths are in parallel, the overall capacitance is dominated by the water channel capacitance. The surface occupied by the percolative channel is a mere 2.4% of the total surface of the protein exposed to the solvent (~7 nm^2^) and hence it may well-accommodate a few water molecules. Any event that interrupts the high dielectric pathway can drastically lower the layer capacitance as this becomes suddenly dominated by the low capacitance path of the protein. The measured responses can be fully accounted for by assuming that this water channel is indeed interrupted upon binding of the *S*-(+)-carvone, while it is unaffected upon binding of the *R*-(−)-carvone, proving, though indirectly, the existence of a probably very subtle conformational change in the pOBP on chiral interaction that dramatically impacts on the complex dielectric properties and is efficiently transduced by the WGOFET.

In conclusion, a minute change in protein layer capacitance is shown to efficiently modulate a water-gated OFET, allowing for chiral differential detection with large enantiomeric discrimination factor and accurate estimation of interaction energies as low as few kilojoules per mole. This is a unique tool with general applicability that allows neutral ligand detection in the picomolar concentration range. The FET binding curves modelling provide information on the electrochemical free energies derived from the FET dissociation constants while the electrostatic component is isolated from the threshold voltage shifts. These can be combined with the chemical free energies gathered from the complex formation in solution, overall providing a comprehensive picture of the energy balances for a surface-bound pOBP-carvone complex undergoing chiral interactions. The computation of the relative decrease in capacitance on binding, associated to the FET binding curves maximum relative current changes, provides strong support to the hypothesis of pOBP undergoing a conformational change on binding of the *S*-(+)-carvone. This study shows also that an ultra-sensitive detection system can be achieved with an organic bio-electronic device fabricated on a flexible substrate with low cost, printing compatible technology.

## Methods

### pOBP expression

F88WH(6) is a pOBP where the wild-type phenylalanine at residue 88 was substituted by tryptophan and a histidine tag (six residues) was attached to the N terminus of the protein. F88WH(6) pOBP was expressed in a bacterial system using the protocol described by Wei *et al*.^46^ A pET5b vector containing the pOBP F88WH(6) sequence was used to transform *Escherichia coli* BL21(DE3) cells. Bacteria colonies were grown overnight in 10 ml Luria-Bertani/Miller broth containing 100 mg l^−1^ of ampicillin. The culture was diluted 1:100 with fresh medium and grown at 37 °C until a bacterial optical density at *λ*=600 nm of 0.7 was reached. At this stage, protein expression was induced by adding isopropylthio-D-galactoside (IPTG) to a final concentration of 0.4 mM. After 2 h at 37 °C, the cells were harvested by centrifugation, resuspended in 50 mM Tris-HCl pH 7.4 and lysed by sonication. After centrifugation, the protein was found to be present in the supernatant. Protein purification was performed by using combinations of chromatographic steps, anionic-exchange resins, followed by gel filtration. Hydrophobic ligands, present in the broth used to grow the bacteria and entering into the protein binding pocket, were removed by a delipidation process at pH 4.5 (ref. 47). The protein was then dialysed against 50 mM sodium phosphate buffer, pH 7.4. The pOBP is characterized by a hydrophobic β-barrel cavity, whose inner surface is ~500 Å^2^ (ref. 13). The proteins, which have an isoelectric point of 4.55 (ref. 48) are negatively charged in deionized water. The (*R*)-(−)- and (*S*)-(+)-carvone enantiomers are perceived as spearmint or caraway odours with human threshold for detection of 30 and 420 nM (ref. 49), respectively. The weight ratio between pOBP and carvone is ~1.5 × 10^2^ while the volume ratio between the β-barrel cavity and the carvone molecule is ~1.5–3.

### Competitive fluorescent binding assay

Emission fluorescence spectra were recorded using a Perkin Elmer LS55 instrument at room temperature, with a 1 cm light path quartz cuvette and 5 nm slits for both excitation and emission. The affinity of binding of pOBP was measured using the fluorescence probe 1-AMA as previously reported^50^. About 1 μM pOBP-F88W solution in 10 mM phosphate buffer, pH 7.4, was titrated with aliquots of 1 mM 1-AMA in methanol to final concentrations of 0.25–8 μM. The probe was excited at 375 nm and the fluorescence spectra were recorded between 400 to 570 nm, monitoring the signals between 480 and 490 nm. The dissociation constant (*K*_1-AMA_) for the OBP/1-AMA complex was calculated using the equation *y*=(*B*max × *x*)/(*K*_1-AMA_+*x*), where *y* is the degree of saturation, *B*max is the number of maximum binding sites, and *K*_1-AMA_ is the dissociation constant^50^. The calculation was run using the software Sigma Plot and a single binding site was considered for the OBP.

The affinity of pOBP-F88W towards *S*-(+)-carvone and *R*-(−)-carvone and 2-phenylethanol was measured in competitive binding assays, using both pOBP and probe at 1 μM as the final concentration. The ligand solutions, with concentrations ranging from 2 to 20 μM, were prepared in 10 mM phosphate buffer pH 7.4 and used for the binding experiments. Dissociation constants of competitors were calculated from the corresponding IC50 values, using the equation: *K*_Sol_=[IC50]/1+[1-AMA]/*K*_1-AMA_, where [1-AMA] is the free concentration of the fluorescence probe and *K*_1-AMA_ is the dissociation constant of the complex OBP/1-AMA.

For *Polistes dominula* OBP1 (Wasp-OBP), the same method was employed using N-phenyl-1-naphthylamine (1-NPN) as fluorescent probe^51^. The probe was excited at 295 nm and emission spectra were recorded between 337–450 nm.

### pOBP-SAM WGOFET device fabrication

PBTTT-C14 was dissolved (7 mg ml^−1^) in a mixture of 1,2-dichlorobenzene and chloroform (9:1). Gold source (S) and drain (D) interdigitated electrodes were photo-lithographically defined on a flexible substrate. The distance between two differently biased fingers is the channel length (*L*), while the perimeter of each set of equipotential fingers is the channel width (*W*); they are 5 μm and 10^4^ μm, respectively. The substrate with patterned electrodes was spin coated with a PBTTT-C14 solution at 7,000 r.p.m. for 60 s and annealed at 120 °C for 10 min. A gold plate with an area of ~3 10^−2^ cm^2^ served as the gate (G) electrode. The SAM functionalizing protocol^52^ involved a 50 mM solution of 3-mercaptopropionic acid (3MPA) in ethanol containing 5% acetic acid. N_2_ was bubbled through the 3MPA solution for at least 10 min to remove dissolved oxygen. The gold electrodes were immersed into the 3MPA solution and kept in the dark under N_2_ for 18 h at 22 °C. The 3MPA SAM obtained had a height of 0.42 nm (ref. 52). For the bio-functionalization protocol, the SAM was activated by immersion into a 100 mM 1-ethyl-3-dimethylaminopropylcarbodiimide hydrochloride and 200 mM N-hydroxysuccinimide aqueous solution for 1 h at 25 °C. Finally, the pOBPs (0.7 mg ml^−1^ in 20 mM Na phosphate buffer, pH 8.0) were left to immobilize for 2 h at 25 °C. Electrochemical inspection of the Au-electrode showed that after the bio-functionalization the electrode surface was passivated (Supplementary Fig. 2 and Supplementary Fig. 3). X-ray photoelectron spectroscopy analysis showed that the appearance of nitrogen and sulfur peaks is only seen on the bio-functionalized gate (Supplementary Fig. 4, Supplementary Fig. 5 and Supplementary Fig. 6). Also the scanning electron microscope micrographs of the pOBP-SAM gold surface showed a very compact and smooth morphology clearly different from that of the Au-surface (Supplementary Fig. 7a,b).

### WGOFET measurement of pOBP-carvone binding curves

The pOBP-SAM gate was brought into contact with a previously dispensed 3 μl of deionized water droplet covering the OSC (Fig. 1a). The electrical characterization of the WGOFET was carried out by a semiconductor parameter analyzer. For the output characteristics, the drain current (*I*_DS_) was measured as a function of the drain voltage *V*_DS_ at gate voltages *V*_GS,_ ranging between 0 and −0.5 V, in steps of −0.1 V. The curves were measured in the forward and reverse mode. For the transfer characteristics *I*_DS_ was measured as a function of *V*_GS_ (+0.02 to −0.5 V) at *V*_DS_=−0.5 V. The device current was stabilized by cycling the measurement of the transfer curve until the overlap of subsequent traces was obtained. This process leads to the filling of the OSC low-mobility trap states leading to a stable *V*_T_ value^17^. After stabilization, the pOBP-SAM gate was incubated for 5 min in a carvone solution and the transfer characteristic was recorded. The Δ*I*/*I* is the electronic response at a given concentration and the relevant dose–response curve is obtained by plotting these data points at all investigated concentrations as the average values over three replicates on different devices, with the relative error taken as 1 s.d.

## Author contributions

M.Y.M. and M.M. performed the FET measurements while E.T. worked on the expression and purification of the OBPs and the complex formation measurements in solution. G.L. carried out the theoretical modelling of the capacitance while G.P. worked on the thermodynamics of the FET system. K.P. supervised all the work connected with the pOBP production and binding in solution. L.T. supervised the work performed on the FET and wrote the manuscript that was revised by all the authors.

## Additional information

**How to cite this article:** Mulla, M. Y. *et al*. Capacitance-modulated transistor detects odorant binding protein chiral interactions. *Nat. Commun.* 6:6010 doi: 10.1038/ncomms7010 (2015).

## Supplementary Material

Supplementary Information Supplementary Figures 1-8, and Supplementary Reference

## Figures and Tables

**Table 1:** Standard Gibbs free energies for the *S*-(+) and *R*-(−) carvones binding to the pOBP.

	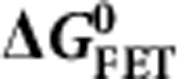 **(kJ mol**^−1^**)**	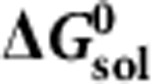 **(kJ mol**^−1^**)**	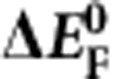 **(kJ mol**^−1^**)**	***W*** **(kJ mol**^−1^**)**
*S*-(+)	−(49.2±0.1)	−(36.00±0.05)	−(5.8±0.5)	−(7.2±0.5)
*R*-(−)	−(41±2)	−(33.0±0.1)	−(1.1±0.5)	−(7.9±2)
Δ*G*^0+^−Δ*G*^0−^	−(7±2)	−(3.0±0.2)	−(4.7±1)	

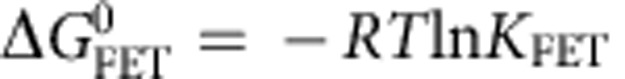
 is estimated from the WGOFET binding curves of Fig. 5b; 
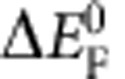
 is the molar electrostatic energy evaluated from the WGOFET *V*_T_ shift; 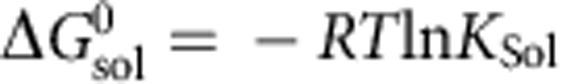 is the binding free energy of the pOBP dissolved in solution; 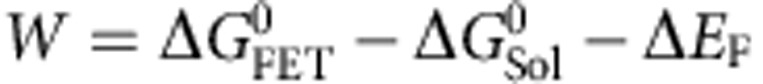 is the surface binding work.
