# Supplementary material for: Capacitance-modulated transistor detects odorant binding protein chiral interactions
Source: Nat Commun. 2015 Jan 16;6:6010. doi: 10.1038/ncomms7010 (PMC4309438; doi:10.1038/ncomms7010)
Supplement: Supplementary Information — Supplementary Figures 1-8, and Supplementary Reference [file ncomms7010-s1.pdf]

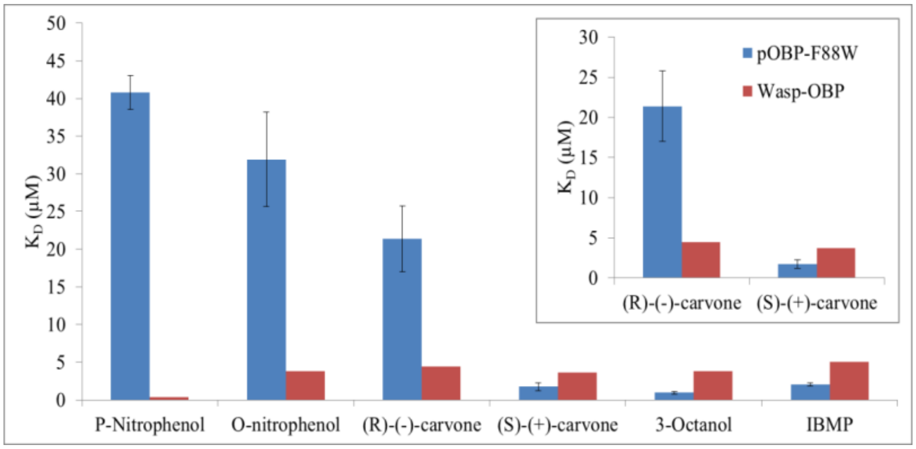

Supplementary Figure 1. Dissociation constants (K_{Sol}) obtained for different analytes for non-delipidated (porcine) pOBP-F88W and (Polistes dominula) Wasp-OBP. Inset shows comparison between obtained values of K_{sol} for the two Carvone enantiomers titrated against pOBP-F88W and Wasp-OBP. The dissociation constants for different ligands, p-nitrophenol, o-nitrophenol, (S)-(+)-carvone, (R)-(-)-carvone, 3-Octanol, 2-isobutyl-3-methoxypyrazine (IBMP), against non-delipidated pOBP-F88W and Wasp-OBP were determined by competitive binding assay. The water soluble ligands (p-nitrophenol, o-nitrophenol) were directly dissolved in water while the other ligands stock solutions were prepared in dimethyl sulphoxide (DMSO) or methanol wherever required and further dilutions were carried out in aqueous solutions. The pOBP-F88W responded differently to the two enantiomers of carvone while no significant difference in the affinities was observed by wasp-OBP (Note: insect OBPs have different tertiary structures from mammalian OBPs). The results confirm that even non-delipidated OBPs show the same order of differential selectivity to carvone enantiomers. If the proteins are delipidated after purification, this enhances the binding affinity while maintaining the selectivity of ligands by the OBPs.

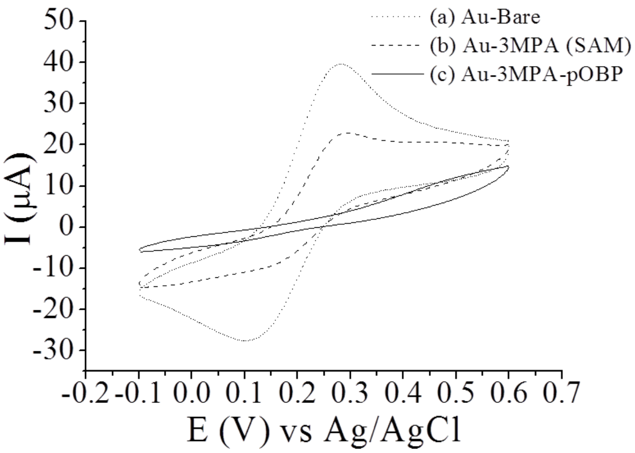

Supplementary Figure 2. Cyclic voltammograms measured in 1 mM $K_4Fe(CN)_6$ supporting electrolyte prepared in 0.1 M KCl for (a) Bare Au electrode (b) Au after functionalization with 3-mercaptopropionic acid (3MPA) SAM and (c) Au-3MPA functionalized with pOBP-F88W. The cyclic voltammograms were recorded using an electrochemical analyzer (Model CH-1140B) from CH instruments Inc. using an Au bare electrode (a), the Au electrode modified with the 3-mercaptopropionic acid (3MPA) SAM and (b) the Au with the SAM and pOBP (c). The supporting electrolyte was 0.1 M KCl in which 1 mM of the $K_4Fe(CN)_6$ electro-active compound was dissolved. The potential was scanned between -0.1 V to +0.6 V Vs Ag/AgCl reference electrode with scan rate of 0.1 V/S. The gold electrodes served as the working electrode while a platinum plate served as a counter electrode. The bare Au electrode (a) shows a typical CV curve with distinct oxidation and reduction peaks with higher oxidative (Io) and reductive (IR) peak currents compared to those of functionalized electrodes (b) and (c). Reduction in I_0 and IR is observed after SAM immobilization (b) and negligible oxidation and reduction peaks are observed after complete functionalization of electrode with pOBP-F88W (c).

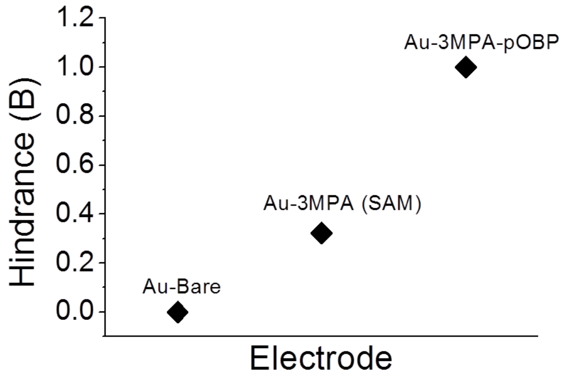

Supplementary Figure 3. Surface hindrance calculated for (a) bare Au electrode (b) Au after functionalization with 3-mercaptopropionic acid (3MPA – SAM) and (c) Au-3MPA-SAM electrode functionalized with pOBP-F88W. The qualitative value for layer density is determined by calculating the steric hindrance coefficient B, given by the formula $B = 1 - (I_0^{Fun} / I_0^{Au})$, where, I_0^{Fun} and I_0^{Au} are the oxidative peak currents obtained from the CV curves for functionalized electrode (3MPA alone and 3MPA-pOBP) and the bare Au electrode respectively. The value of B increases with the number of layers on the gold gate electrode reaching maximum value of 1 for a completely functionalized electrode confirming passivation and hence complete coverage of gate electrode.

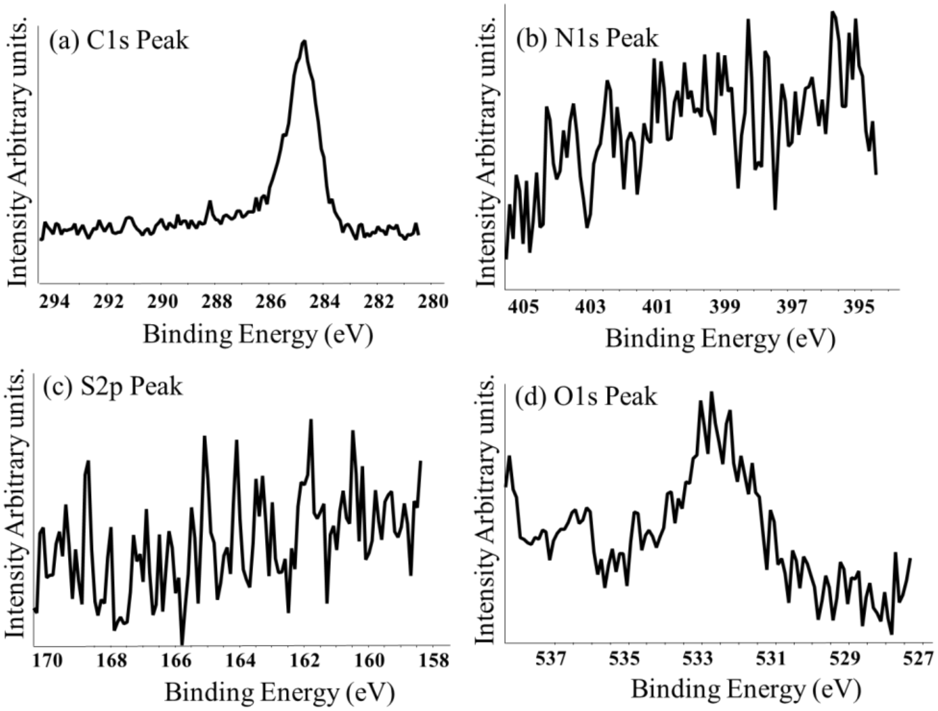

Supplementary Figure 4. High resolution XPS spectra of bare gold electrode. All X-ray photoelectron spectroscopy (XPS) measurements were performed with a Theta Probe Thermo-VG Scientific instrument using an Al Kα monochromatic source (15 kV with 300 μm X-ray spot size and take-off angle of 37°, the base pressure was maintained at 10^{-9} mbar). Survey spectra (0 - 1200 eV binding energy, BE) and high - resolution spectra were acquired at 150 and 100 eV pass energy, respectively. Data was analyzed using the Thermo Avantage software, version 4.75. A C1s spectrum was taken as a reference for all elements. Charge neutralization was used when substrates showed artifacts arising from surface charging effects. The bare gold electrode shows no detectable nitrogen and sulfur compounds ascribable to the protein and to the 3MPA functionalities. The XPS spectrum show only carbon and oxygen signals due to atmospheric hydrocarbon contamination.

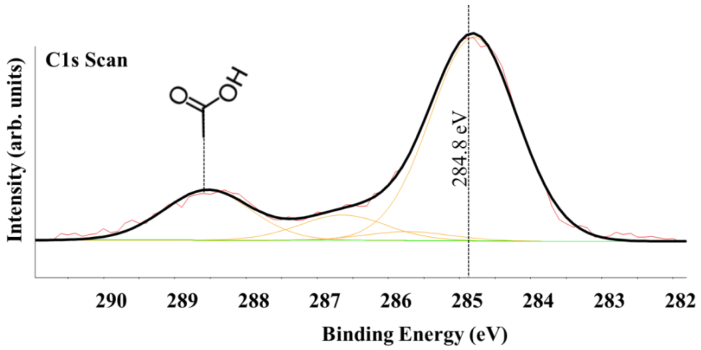

Supplementary Figure 5. High resolution XPS spectra of Carbon for Au electrode functionalized with self-assembled monolayer of 3-mercaptopropionic acid (3MPA-SAM). The characteristic carboxylic functionality is shown at higher binding energy around 289 eV. The XPS analysis of the 3MPA-SAM functionalized gate electrode confirmed the successful 3MPA-SAM formation on gold surface with characteristic carboxylic groups observed at higher binding energy of around 289 eV. A sulfur peak was observed while no detectable nitrogen was observed in case of only 3MPA-SAM functionalized electrode.

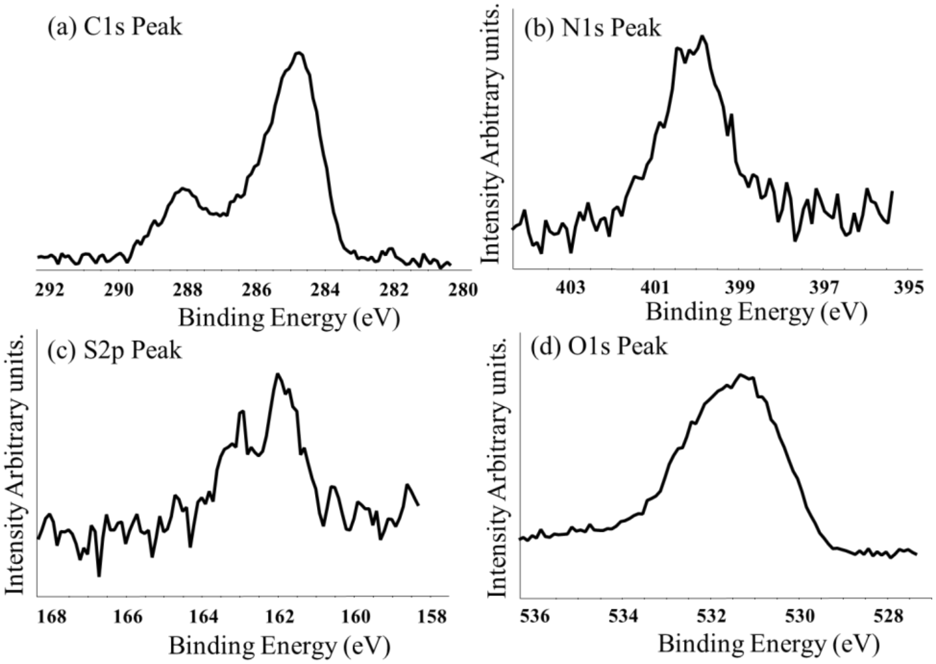

Supplementary Figure 6. High resolution XPS spectra of pOBP-SAM functionalized gold electrode confirming presence of (a) carbon 1s, (b) nitrogen 1s, (c) sulfur 2p and (d) Oxygen 1s. The data acquired for the gate electrode functionalized with the pOBP-SAM, additionally show both the N1s and S2p peaks. The presence of nitrogen compounds proves the successful attachment of the pOBP-F88W to the electrode surface. The presence of the sulfur peak is attributed to chemical functionality from the pOBP-SAM and Au-S bonds of immobilized 3MPA-SAM.

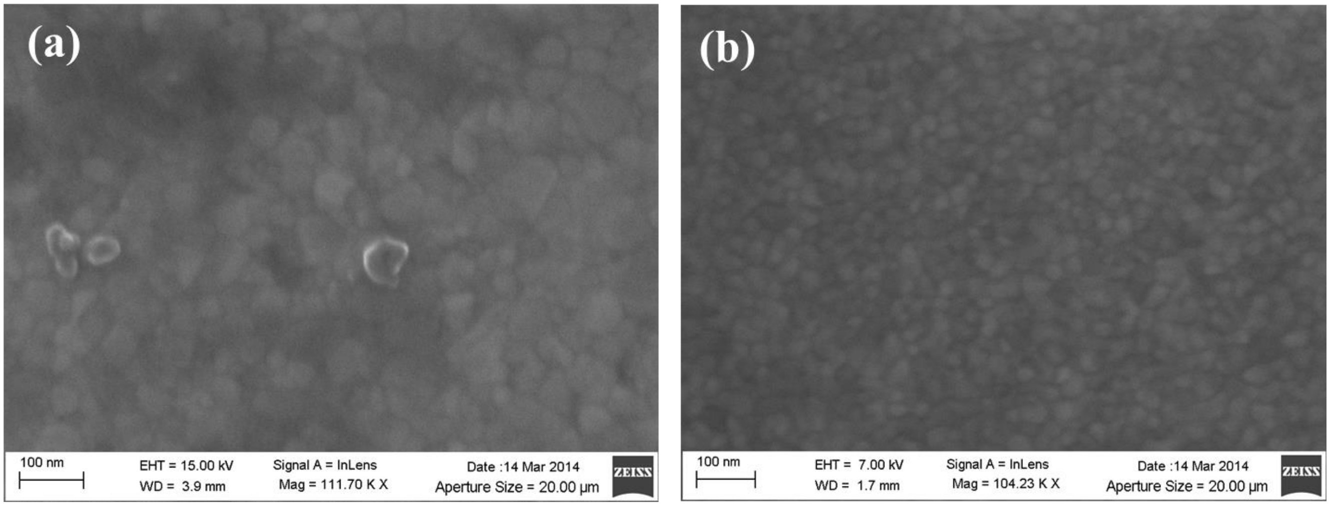

Supplementary Figure 7. **(a)** SEM image of a pOBP-SAM functionalized gold substrate. **(b)** Bare gold electrode showing uniformly distributed gold grain. Scanning electron microscopic (SEM) analysis was performed by Carl Zeiss SEM (Sigma model). The SEM investigation was performed on Si/SiO_2 substrates covered by a thermally evaporated gold, 50 nm thick with 5 nm Ti adhesion promoting layer. The surface morphological features of bare gold surface and the same surface after pOBP-SAM bio-functionalization are shown. In the protein modified surface features of few tens of nm can be seen that are ascribable to a very compact pOBP-SAM formed of protein aggregates.

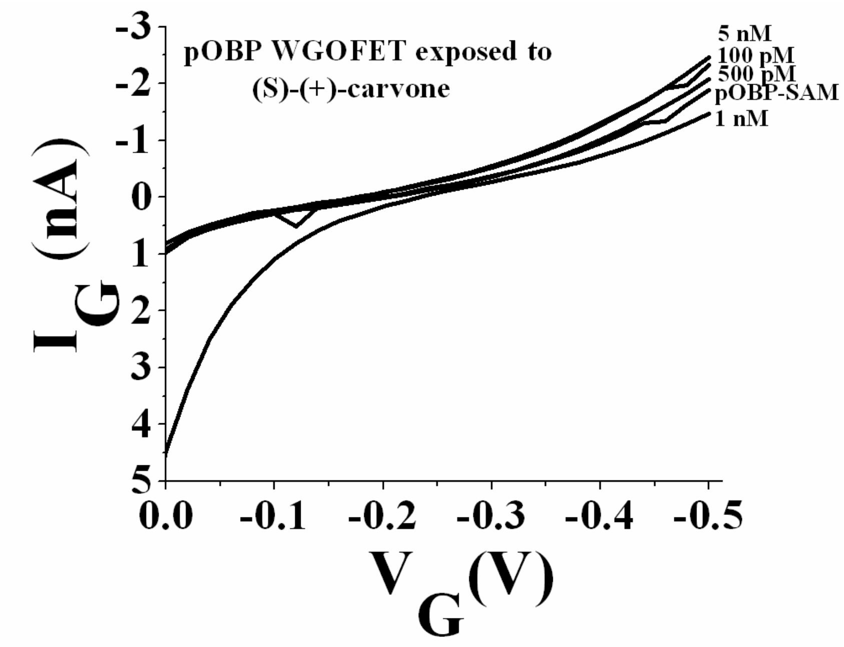

Supplementary Figure 8. The gate leakage current (I_G) as a function of the gate bias (V_G) for the pOBP-WGOFET exposed to different concentrations of (S)-(+)-carvone. The gate leakage current (I_G) is reported as a function of the gate bias. The data are measured contemporarily to the transfer characteristics reported in the main text as Figure 5a. The important point to outline is that I_G is almost three orders of magnitude lower than $I_{DS.}$ The I_G traces measured are therefore less stable. Their change upon exposure to the ligand analyte is very scattered as far as the scaling with the analyte concentration is concerned. This experiment clearly proves the necessity to use the field-induced current I_{DS} as signal to perform a reliable and sensitive analysis.

Supplementary Reference

1. Weisser, M., Nelles, G., Wohlfart, P., Wenz, G. & Mittler-Neher, S. Immobilization kinetics of cyclodextrins at gold surfaces. *J. Phys. Chem.* **100**, 17893-17900 (1996).
